# Ovariotomy by Enucleation

**Published:** 1873

**Authors:** Samuel Logan, W. H. Ford


### OVARIOTOMY BY ENUCLEATION.

REPORTED BY SAMUEL LOGAN, M.D.,* AND W. H. FORD, M.D.†

Prof. Miner's plan of operating for ovarian tumor, being still *sub judice*, it seems to be the duty of all who resort to that method to report their results, and under that conviction we record the following case, the early symptoms of which were developed under the immediate observation of Prof. Ford, in whose practice the case occurred.

Mrs. A. H. L., aged forty-two; married; multipara; nervous and excitable, subject to hysterical paroxysms; has not menstruated since birth of last child, now twenty-one months old.

*Professor of Anatomy and Clinical Surgery in the University of Louisiana.
†Professor of Physiology in New Orleans School of Medicine.

*May* 12, 1872.—Has a tumor in hypogastric region as large as the uterus at four months utero-gestation, ovoidal in shape, movable.

*June* 8*th.*—Tumor increased in size until it now fills the abdominal cavity; everywhere tender on pressure, or even to the slightest touch. Dullness on percussion over abdomen; fluctuation in the neighborhood of umbilicus. General contour of tumor clearly recognizable; vagina and cervix normal; uterus immovable in hollow of sacrum. No fœtal heart-sound, but a constant murmur closely simulating the placental suffle.

*June* 9*th.*—Patient in great distress; demanding relief; abdomen tense and extremely painful to touch; fluctuation perceptible over the whole abdomen. Pains, similar to those of labor, coming on every hour or two. The uterine sound could not be introduced more than two and a half inches. Under diet, warm water douches in the vagina, stramonium poultices to abdomen, etc., the acute symptoms ceased after a few days.

The general fluctuation, the presence of bosselated enlargements on the sides of the pyriform mass, the rapid growth, and acute pains, determined the diagnosis in favor of cystic degeneration of the wall of the uterus, or of some of the annexes of that organ.

*June* 15*th.*—Chloral at night; abdominal pains, especially severe on turning in bed. Bowels regular; urine very scanty and high-colored. Fever from time to time, but more in the last two days. Pain in the right iliac region. Appetite and digestion good.

*June* 25*th.*—During last forty-eight hours has had a dribbling of clear watery fluid from vagina. Tapped in linea alba one and a half inches below umbilicus, and one and a half pints of glairy, flocculent, citrine fluid escaped during an hour; and as much more during the ensuing thirty-six hours, when the wound was closed with a bit of strap. Relief decided; very slight inflammation about the puncture. Ordered quinia and iron.

After this, patient was tapped seven times, at intervals of from five weeks to six or eight days. The last tapping on October 10th. Fluids evacuated in all cases similar; citrine, glairy, and, towards the close of the tapping, almost puriform. Viscidity most marked in fluids obtained from the harder nodular masses of the left side cf the body of the tumor. Punctures gave no trouble. The quantity of fluid drawn off at each tapping varied from three pints to two gallons. The puncture made in the last tapping was intentionally kept open by the patient, in view of the relief from oppression, now very urgent, afforded even at the inconvenience of the constant discharge. Notwithstanding the escape of so much fluid, secretion was so rapid that enlargement continued. Puffiness under the eyes; legs and feet œdematous and cold; appetite fair; digestion imperfect; colicky pains; pulse eighty-five. Girth through umbilicus, thirty-four inches; from pubis to ensiform cartilage, fifteen inches.

*November* 10, 1872.—Ovariotamy having been decided on, it was performed this day by Prof. Logan, assisted by Prof. Ford, and by Drs. A. H. Cage, C. B. Galloway, Jr., of Canton, Miss. The patient having been put under the influence of chloroform, incision was made, extending from the point at which the last tapping was performed, and from which the discharge was still issuing, about an inch below the umbilicus to the symphysis pubis. The opening into the peritoneal cavity was commenced below, and extended upwards, so as to be certain not to cut into the tumor, which there was every reason to believe was adherent round the orifice of the last tapping. As a rule, it is advisable to open below when the above condition is not present. The interval between the peritoneum and the tumor is much more easily found below, where the abdominal walls are reflected from the margin of the tumor to the pubes, than above, where tumor and abdominal walls are closely applied. When the peritoneum was split open, the expected adhesions were found to exist, but they were easily torn loose. Spencer Wells' large hollow trocar and canula, with gutta-percha tube attached, was then plunged into the tumor through the fistula; but so very much softened had the adjacent portions of the cyst-wall become, that it tore like wet paper, permitting the glairy and semi-purulent fluid to flow over the tumor. This complication was promptly met, however, by pressure applied to the lateral abdominal walls covering the tumor, which effectually guided the wave of fluid through the lips of the wound. By this prompt action but little of the escaping fluid entered the cavity of the peritoneum. Most of the cystic contents were evacuated in this way. An immense quantity was thus expressed, most of the other large cysts seeming to communicate with this opening. Indeed, at the last tapping a long canular and trocar had been used and projected in several directions, with the view of effecting just such a communication in order to make the tapping the more effective. After the fluid ceased to flow, the usual exploration was made, and the tumor was found tightly and extensively adherent to the abdominal walls on each side. The mass was still so large that it became at once evident that an extension of the incision in the abdominal walls would be required. The incision was, therefore, at once continued upwards and around the umbilicus to about two inches above that point. It was then found that there was also one point of adhesion to the omentum. This was easily separated, and so were the far more extensive lateral connctions already mentioned. In performing this part of the operation, particular care was taken to effect the separation at the expense of the cyst-wall, rather than the normal tissues; and the separation was effected with much less trouble than had been anticipated. The tumor was then turned out of the abdomen, and found to be connected with the right broad ligament, by means of a pedicle about two inches broad and about three-quarters of an inch thick. It was quite long enough for clamping, and one of Mr. Spencer Wells' clamps was provided, in case enucleation, which had been determined on, was found inadvisable. Insinuating the index finger through the middle of the pedicle where it joined the tumor, the operator succeeded with perfect ease in carefully peeling each portion with its vessels from the surface of the former, and in a very short time the whole mass was everted, without the loss of a half drachm of blood, and the shreddy pedicle was dropped back into the abdomen. There was some little hemorrhage during the operation, but it was mostly venous and from the abdominal walls, the veins in that position having been considerably distended, probably from the pressure of the tumor on the ascending cava. What fluid and blood had settled into the pelvic and abdominal cavity were carefully sponged out. The womb, the remaining ovary, and the other parts were examined and found perfectly healthy; and the wound was closed by silk sutures extending through all the thickness of the abdominal parieties. The line of incision was then glued up with Richardson's colloid styptic; the abdominal walls were supported with long strips of adhesive plaster running across the wound, and extending well round the flanks, and the line of incision was covered with a piece of lint soaked in carbolic oil (one part carbolic acid to seven of olive oil.)

The patient was then conveyed from the operating table and placed on her back in bed.

She recovered readily from chloroform, and did not seem to suffer any marked degree of surgical shock. Pulse, one hour after operation, 120; skin almost normal; mental condition natural.

Tumor weighed, after evacuation of fluid, 16 lbs.; estimated weight of fluid lost during operation, say 8 lbs.; total estimated weight, 24 lbs. Examination by microscope and otherwise shows usual structure of the multilocular ovarian tumor.

The patient progressed favorably. On the tenth day the stitches were removed; union firm along the whole line of incision, except at one point, where a little suppurative action had occurred. An alum wash reduced this in a day or two. An abdominal waistcoat was applied to support the line of adhesion.

*December 1st.*—Progress very rapid and uncomplicated; patient sat up on the thirteenth day in bed, and was about her room on the eigteenth day. Afterwards continued to improve on cod-liver oil, quinia, and iron. A dull pain in the lower abdomen, felt after the operation, disappeared by degrees. She fattened remarkably and on the fortieth day menstruated.

At the present writing—more than four months since the removal of the tumor—she is in perfect health.—*Amer. Jour. of Med. Science, and Richmond & Louisville Med. Jour.*

---

Ergot of Rye.—In France, by a decree dated June 23, 1873 the sale of ergot of rye, previously placed in the list of poisons, and which could only be used in medicine by druggists on the prescription of a physician, surgeon, officer of health, or veterinarian, can now equally be dispensed by druggists on the prescription of a midwife furnished with a diploma.—*Gaz. des Hôpitaux, June 28th*, 1873.

